# Neutrophil extracellular traps-mediated Beclin-1 suppression aggravates atherosclerosis by inhibiting macrophage autophagy

**DOI:** 10.3389/fcell.2022.876147

**Published:** 2022-07-18

**Authors:** Masataka Sano, Yasuhiro Maejima, Shun Nakagama, Yuka Shiheido-Watanabe, Natsuko Tamura, Kenzo Hirao, Mitsuaki Isobe, Tetsuo Sasano

**Affiliations:** ^1^ Department of Cardiovascular Medicine, Tokyo Medical and Dental University (TMDU), Tokyo, Japan; ^2^ Department of Professional Development, Tokyo Medical and Dental University Hospital, Tokyo, Japan; ^3^ Department of Cardiovascular Medicine, AOI Universal Hospital, Kawasaki, Japan; ^4^ Sakakibara Heart Institute, Tokyo, Japan

**Keywords:** Beclin 1, autophagy, neutrophil extracelluar traps, macrophage, atheroscelrosis

## Abstract

A growing body of evidence suggests that neutrophil extracellular traps (NETs) critically contribute to the development of atherosclerosis. However, the detailed mechanism of how NETs promote atherogenesis remains unknown. In this study, we explored the role of NETs for promoting atherosclerosis by modulating the activity of autophagy in macrophages. NETs were effectively induced by a nicotine administration to the HL-60 cell-derived neutrophil-like cells. Treatment with NETs markedly suppressed both autophagosome formation and autophagosome–lysosome fusion in 7-ketocholesterol-treated macrophages, which are accompanied by the enhancement of inflammasome activity. NETs upregulate epidermal growth factor receptor (EGFR) activity, which enhances Beclin-1 phosphorylation of the tyrosine residues of Beclin-1 by EGFR, inhibits the PI3 kinase activity of the Beclin1–Vps34 complex, and suppresses autophagosome formation in macrophages. Furthermore, NET-induced activation of EGFR allows Rubicon to increase its expression, thereby suppressing autophagosome-lysosome fusion. *In vivo* experiments revealed that the suppression of NET formation by ablating peptidyl arginine deiminase-4 in neutrophil leukocytes resulted in the attenuation of atherosclerotic plaques in a nicotine-administered HFD-fed *ApoE* ^*−/−*^mice. Taken together, these results suggest that NET-mediated EGFR–Beclin-1 signaling in the macrophages promotes atherogenesis by autophagy inhibition-mediated inflammasome activation.

## Introduction

Atherosclerosis causes lethal consequences including the stroke and coronary artery diseases (Roth et al., 2020). Growing body of evidence suggest that the atherosclerotic lesions are developed under the predisposing conditions including hyperlipidemia, hypertension, diabetes, and smoking habits, which result in the inflammatory response of the arterial walls. However, it remains unknown that through the precise molecular mechanisms how to induce the atherosclerosis through a detrimental stimuli-induced inflammatory response.

Neutrophil Extracellular Traps (NETs) are immunoregulatory mechanisms that are formed when the peptidyl arginine deiminase-4 (Pad4) of neutrophil leukocytes is activated in response to an external stimuli, which causes induction of a nuclear histone citrullination, thereby promoting the chromatin release into the extracellular space (Li et al., 2010). NETs include enzymes such as neutrophil elastase (NE), cathepsin-G, myeloperoxidase (MPO), and nucleosomes, a complex of DNA and citrullinated histones (Yang et al., 2016). Actually, NETs are discovered as one of the host defense systems for disarming and killing bacteria in the extracellular spaces (Brinkmann et al., 2004). On the other hand, increasing lines of evidence suggests that the NETs play an important role in the induction and progression of the various immune-mediated diseases such as rheumatoid arthritis, bronchial asthma, malignant neoplasms (Papayannopoulos, 2018), and atherosclerosis (Warnatsch, Ioannou, Wang and Papayannopoulos, 2015). Indeed, previous preclinical studies demonstrated that the suppression of the NETs formation significantly reduced the asprogression of atherosclerosis in *ApoE*-deficient (*ApoE* ^*−/−*^) mice (Knight et al., 2014; Jiménez-Alcázar et al., 2017; Franck et al., 2018). However, the details regarding which NETs are activated by any of the stimuli to develop and progress the atherosclerotic lesions and plaque instability remains unknown.

Autophagy is an intracellular mechanism by which the aggregated proteins and damaged organelles are enveloped in a lipid bilayer called autophagosome and transported to lysosomes for a removal (Mizushima, Levine, Cuervo and Klionsky, 2008). Autophagy plays a role in maintaining the cellular homeostasis and regulating the defense responses to stress, where it plays a protective role by controlling the quality of the intracellular proteins and removing danger signals (Sciarretta, Maejima, Zablocki and Sadoshima, 2018). One of the critical regulators of autophagy is Beclin-1, a mammalian ortholog of the Atg6 in yeast (Liang et al., 1999). Beclin-1 forms three distinct molecular complexes: Beclin-1-Vps34-Vps15-Atg14L complex, Beclin-1-Vps34-Vps15-UVRAG complex, and Beclin-1-Vps34-Vps15-UVRAG-Rubicon complex (Matsunaga et al., 2009; Rong et al., 2009). Among them, different from the other two complexes, a Beclin-1 complex containing Rubicon plays a suppressive role in the regulation of the autophagosome–lysosome fusion, the final step in autophagy, which is mediated by SNARE proteins, including Vamp8 and Syntaxin-17 (Itakura et al., 2012; Nah et al., 2021). Specifically, Rubicon negatively regulates the autophagosome–lysosome fusion by the sequestration of UVRAG from the Beclin-1-associated complex, which results in the attenuation of Vps34 kinase activity (Matsunaga et al., 2009; Zhong et al., 2009). The activity of Beclin-1 is tightly regulated by various post-translational modifications such as phosphorylation (Maejima, Isobe and Sadoshima, 2016). We and other investigators have shown that the serine/threonine kinases phosphorylate Beclin-1, thereby modulating the autophagy machinery by regulating the Beclin-1-associated class III PI3 kinase activity (Wei, Pattingre, Sinha, Bassik and Levine, 2008; Zalckvar, Berissi, Eisenstein and Kimchi, 2009; Fogel et al., 2013; Maejima et al., 2013). Beth Levine and her groups discovered that the activated epidermal growth factor receptor (EGFR) phosphorylates multisite tyrosine residues of Beclin-1 in Tyr229, Tyr233, and Tyr352, which decreases its function and suppresses autophagy (Wei et al., 2013). Such a discovery revealed the molecular link between an oncogenic receptor tyrosine kinase and the core autophagy machinery that critically contributes to tumor progression in cancer cells.

Increasing evidence reveals that autophagy in the macrophages plays a critical role in alleviating the atherosclerosis by degrading inflammasome, a molecular complex that provokes the inflammatory response due to an innate immunity (Liao et al., 2012; Razani et al., 2012; Ito et al., 2021). In addition, it has been shown that the NETs license macrophages for inflammasome activation, thereby developing the atherosclerotic lesions (Kolaczkowska et al., 2013). Furthermore, it has been shown that NETs inhibit autophagy in the macrophages and other cells (Warnatsch et al., 2015).

Based on such previous findings, we hypothesized that NETs would aggravate the atherosclerosis by modulating autophagy machinery and innate inflammatory response in the macrophages. In the present study, we investigated the mechanism of how NETs regulate macrophage autophagy for the development of atherosclerosis in a hyperlipidemic *ApoE* ^*−/−*^mice.

## Materials and methods

### Experimental animals

We used 12-week-old male mice of *C57BL/6J* wild-type (WT) (CLEA Japan, Tokyo, Japan), *ApoE* ^*−/−*^(#002052), *Pad4-floxed* (*Pad4* ^*fl/fl*^; #026708) and *Mrp8-Cre* transgenic (*Tg-Mrp8-Cre*; #021614) (Jackson Laboratory, Bar Harbor, ME, United States). To generate a neutrophil-specific Pad4-deficient *ApoE* ^*−/−*^(*ApoE* ^*−/−*^; *Neu-Pad4* ^*−/−*^) mice, *Pad4* ^*fl/fl*^ mice were crossed with *Tg-Mrp8-Cre* mice, and then crossed them with an *ApoE* ^*−/−*^mice. These animals were housed in a pathogen-free animal care facility under the standard laboratory conditions (27°C, 40%–60% humidity, 12-h light/12-h dark cycle) and allowed full access to fresh water and a standard rodent chow (CLEA Japan, Tokyo, Japan). *ApoE* ^*−/−*^mice and *ApoE* ^*−/−*^; *Neu-Pad4* ^*−/−*^mice were fed a high-fat diet (HFD) of Western type containing 21% pork lard and 0.15% cholesterol (CLEA Japan, Tokyo, Japan) with or without a nicotine tartrate dihydrate (24303-84, Nakalai Tesque, Kyoto, Japan) in drinking water (100 μg/ml) for 20 weeks. All the animal care and experimental procedures were approved by the Tokyo Medical and Dental University Guide for the Care and Use of the Laboratory Animals (Permit Number: A2021-023A), and the Guide for the Care and Use of the Laboratory Animals published by the US National Institutes of Health.

### Generation of neutrophil extracellular traps and their purification

HL-60 cells (ATCC: CCL-240) were cultured in the RPMI-1641 (Sigma-Aldrich, St. Louis, MO, United States) supplemented with the 10% fetal bovine serum (FBS) and 1% penicillin/streptomycin (P/S) and maintained them at 37°C in a 5% CO_2_ incubator. To differentiate HL-60 cells into a neutrophil-like cells, a total of 200 µM of all-trans retinoic acid (ATRA, #186-01114, Fujifilm-Wako, Tokyo, Japan) was treated into HL-60 cells and incubated them for 72 h, as described previously (Kawakami et al., 2014). NETs were isolated as previously described with a minor modification (Najmeh, Cools-Lartigue, Giannias, Spicer, and Ferri, 2015). Briefly, 50 µM of phorbol 12-myristate 13-acetate (PMA, #P8139, Sigma-Aldrich, St. Louis, MO, United States) or 10 µM of the nicotine was added to the HL-60-derived neutrophil-like cells and incubated for 2 h. After aspirating the supernatant off, the surface of the culture dishes was gently washed with 5 ml of phosphate-buffered saline (PBS). The collected samples with the PBS were spun down at 15,000 rpm × 5 min, then the pellet was resuspended in 100 µl of PBS after removing the supernatant. The induced NETs were determined by the immunofluorescence, scanning electron microscopy, and immunoblotting. After determining the NET DNA concentration as previously described (Warnatsch et al., 2015), the concentration of NETs was adjusted to 500 µg/ml by adding PBS for the co-culture experiments.

### Co-culture experiments of macrophages and neutrophil extracellular traps

THP-1 cells (ATCC: TIB-202) were cultured in RPMI-1641 (Sigma-Aldrich, St. Louis, MO, United States) supplemented with the 10% FBS and 1% P/S and maintained them at 37°C in a 5% CO_2_ incubator. To differentiate the THP-1 cells into macrophages, a total of 50 nM of PMA was treated to THP-1 cells and incubated them for 48 h. Autophagy was induced by adding the 70 µM of 7-ketocholesterol (7KC). To evaluate the effect of the NETs on macrophages, a total of 5 or 50 µg of NETs were added with or without a treatment with the EGFR tyrosine kinase inhibitor AG1478 (#S2728, Selleck Chemicals, Houston TX, United States), and then the samples were collected after 30 min or 4 h.

### Immunoblot analyses

Cultured cells were lysed in the lysis buffer [1% Triton X-100 (Sigma-Aldrich), 50-mM Tris, 150-mM NaCl, and 2-mM EDTA diluted with distilled water], phosphatase inhibitor cocktail tablets (PhosSTOP EASYpack; Roche Diagnostics, Indianapolis, IN, United States), and protease inhibitor cocktail (Sigma-Aldrich) on ice for 10 min. The mixture was centrifuged at 15,000 rpm for 10 min, and the supernatant was analyzed *via* immunoblotting. Equal amounts (10 µg per lane) of the total proteins from each lysate were separated by a sodium dodecyl sulfate–polyacrylamide gel electrophoresis and transferred into nitrocellulose membranes, which were incubated with the primary antibodies, washed, and then reacted with the secondary antibodies [HRP-linked anti-rabbit IgG (#7074) or HRP-linked anti-mouse IgG (#7076); Cell Signaling] at a room temperature. Specific signals were visualized *via* chemiluminescence using the SuperSignal West Dura Extended Duration Substrate (Thermo Scientific, Waltham, MA, United States), and immunoreactive bands were visualized by using the quantified iBright1500 imaging system (Thermo Scientific, Waltham, MA, United States). Primary antibodies against the following proteins were used: LC3 (#M186-3; MBL, Nagoya, Japan), p62/SQSTM1 (#PM045; MBL), NLRP3 (#A017374 Atlas Antibodies, Bromma, Sweden), Cleaved IL-1β (Asp116) (#83186; Cell Signaling), Beclin-1 (#3738; Cell Signaling), NE (#ab131260; Abcam), Citrullinated Histone H_3_ (#ab5103; Abcam), EGFR (#4267; Cell Signaling), phospho-EGFR (Tyr1068) (#3777; Cell Signaling), *DYKDDDDK* (=Flag, #14793; Cell Signaling), Vamp8 (#ab76021; Abcam), Syntaxin-17 (#ab229646; Abcam), Rubicon (#M170-3; MBL), and β-actin (#4970; Cell Signaling).

### Enzyme-linked immunosorbent assay

The culture media of the THP-1-derived macrophages were analyzed *via* ELISA for the levels of the IL-1β (Human IL-1β ELISA MAX Deluxe Set; BioLegend, San Diego, CA, United States), according to the manufacturer’s protocols. Briefly, the culture media of the THP-1-derived macrophages or standards were added to 96-well plates coated with the capture antibody (anti-IL-1β) and incubated at room temperature for 2 h. The solutions of a detection antibody and Avidin-HRP were sequentially added to the plates, washing and incubating between each adding step. After adding substrate solution to each well of the plates, read absorbance at 450 nm.

### Immunoprecipitation assay

Differentiated THP-1 cells transduced with an adenovirus harboring Flag-Beclin-1 for 48 h with or without treatment with 7KC, NETs (50 µg), and AG1478 centrifuged at 1,000 × g for 10 min, and the pellets were resuspended in 500 µl of lysis buffer (50 mM Tris-HCl, pH 7.4, 1% Triton X-100, 150 mM NaCl, 1 mM PMSF, 10 µg/ml aprotinin, protease inhibitor cocktail, and phosphatase inhibitor) for 10 min on ice. After centrifugation at 15,000 × g for 10 min, the supernatants were incubated with anti-Flag M2 magnetic beads (Sigma, M8823-1ML, Lot# SLBJ 3044V) for 1 h at 4°C, which were then washed three times with 500 µl of Tris-buffered saline with 0.1% Tween 20 at 4°C. The beads were resuspended in a 2 × sodium dodecyl sulfate–polyacrylamide gel electrophoresis (SDS-PAGE) sample buffer, resolved by SDS-PAGE, and analyzed by immunoblotting with an anti-tyrosine phosphorylation antibody (#ab10321; Abcam).

### Evaluation of the whole aortic plaque lesions

Mice were euthanized through an overdose of three types of the mixed anesthetic agents (medetomidine, midazolam, and butorphanol at the concentrations of 0.15, 2.0, and 2.5 mg/kg, respectively). The whole aortas were excised from the carcasses. The whole aortas were opened longitudinally from an ascending aorta to the iliac bifurcation, pinned *en face*, and stained for lipids with the Oil Red O. The stained area was identified as the atherosclerotic lesion area and evaluated as a percentage of the total aortic area by using the ImageJ software (National Institutes of Health, Bethesda, MD, United States).

### Immunocytochemistry

HL-60-derived neutrophil-like cells with or without the PMA or nicotine treatment seeded on the chamber slides were fixed with the 4% paraformaldehyde (PFA) for 10 min. After washing with the PBS and permeabilization with the 1% Triton X-100, the cells were incubated with a blocking buffer [5% bovine serum albumin (BSA), 0.1% Triton X-100] and then with the primary antibodies [Citrullinated Histone H_3_, #ab5103, Abcam; Myeloperoxidase (MPO), #ab208670, Abcam] overnight at 4°C. After washing with the PBS, Alexa Fluor-conjugated (488) secondary antibody was applied with the blocking buffer. Finally, coverslips were mounted on the slides using a Vectashield with 4′,6-diamidino-2-phenylindole (DAPI) (Vector Labs, Burlingame, CA, United States), and photographed.

### Immunohistochemistry

Thin-sliced paraffin-embedded histological sections were processed with antigen retrieval procedures after deparaffinization. To reduce nonspecific binding, sections were incubated with 10% normal bovine serum at room temperature. To stain for macrophages or neutrophil leukocytes, sections were incubated with antibodies against CD11b (#ab128797, Abcam) or MPO (#ab208670, Abcam) at 4°C overnight. After washing with PBS, the tissues were incubated with HRP-labeled polymer anti-mouse antibodies (Dako, K4007) for 30 min at room temperature, and the conjugates were detected using Histofine Simple Stain AEC solution (#H1506, Nichirei Corporation, Tokyo, Japan). Sections were counterstained with hematoxylin. Immunofluorescence double staining for the histological sections was performed to examine the localization of NETs in the atherosclerotic plaques using the same protocol of immunocytochemistry described above. Specimens were observed under confocal microscopy.

### Scanning electron microscopy

The HL-60-derived neutrophil-like cells with or without the PMA or nicotine treatment were fixed with 2.5% glutaraldehyde for 2–5 h, washed overnight in 0.1 M PBS, post-fixed with the 2% OsO_4_ for 2 h, and then dehydrated in the ascending concentrations of ethanol (50%, 70%, 80%, 90%, 95%, and 100%). Then, the specimens were washed with a 3-methylbutyl acetate for 20 min, dried to the critical point, attached to an aluminum mount using a sliver paste, coated with the 7 nm of the titanium, and examined under a scanning electron microscope (S-4500/EMAX-7000, Hitachi) at the different magnifications.

### Transmission electron microscopy

The THP-1-derived macrophages treated with or without a 7KC and/or NETs were fixed in the 2.5% glutaraldehyde in 0.1 M phosphate buffer for 2 h, washed with a 0.1 M PB, post-fixed in a 1% osmium tetroxide in the 0.1 M PB for 2 h, dehydrated in a graded series of ethanol, and embedded in the Epon 812 (TAAB Laboratory Equipment, Aldermaston, United Kingdom). Semi-thin sections were cut at a thickness of 1 μm and stained with a toluidine blue. Ultrathin 90-nm sections were collected on the copper grids, double-stained with the uranyl acetate and lead citrate, and then observed under a transmission electron microscope (H7011; Hitachi High-tech, Tokyo, Japan).

### Phosphatidylinositol 3-kinase assay *in-situ*

To measure the abundance of a membrane-associated phosphatidyl inositol-3-phosphate to evaluate the activity of the Beclin-1-Vps34 complex *in-situ*, we previously generated an Ad-GFP-2×FYVE, which harbors the FYVE (Fab1, YOTB, Vac1p, and EEA1) domain (Maejima et al., 2013). The amino acid sequence we used for this adenovirus construction was as follows: *NH*_*2*_ *-WVPDSQAPNCMKCEARFTFTKRRHHCRACGKVFCASCCSLKCKLLYMDRKEARVCVICHSVL-COOH*. The THP-1-derived macrophages grown on coverslips were transduced with an Ad-GFP-2×FYVE. After 48 h, media were changed into a complete or glucose-free, and cells were treated with or without a 7KC and/or NETs for 2 h. Cells were fixed with a 4% PFA in PBS for 15 min. The number of GFP-2×FYVE dots in the cytoplasm was counted in five independent visual fields.

### Evaluation of fluorescent LC3 puncta

The THP-1-derived macrophages grown on the coverslips were transduced with an adenovirus harboring mRFP-GFP-LC3 (Ad-tf-LC3) at 15 MOI (Hariharan et al., 2010). Twenty-four hours after the adenovirus transduction, the cells were washed with a PBS, fixed with 4% PFA, mounted on the slides using a Vectashield with DAPI (Vector Labs, Burlingame, CA, United States), and viewed under a fluorescence microscope. The number of the GFP and mRFP dots was determined by a manual counting of fluorescent puncta in the five fields from three different cell preparations. The number of dots per cell was obtained by dividing the total number of dots by the number of the nuclei in each microscopic field.

### Statistics

All the statistical analyses were conducted using the GraphPad Prism (GraphPad Software, Inc., San Diego, CA, United States). The normality of distribution was determined using a D’Agostino-Pearson normality test and confirmed that all data were normally distributed. All statistical data are expressed as mean ± standard error of the mean (SEM). All statistical analyses were performed using an unpaired Student’s *t*-test, one-way analysis of variance (ANOVA) or two-way ANOVA, followed by the post-hoc Bonferroni–Dunn method for multiple pairwise comparisons. In all cases, the results were considered statistically significant at a *p*-value < 0.05.

## Results

### Nicotine induces neutrophil extracellular traps that contains abundant neutrophil elastase

**FIGURE 1:**
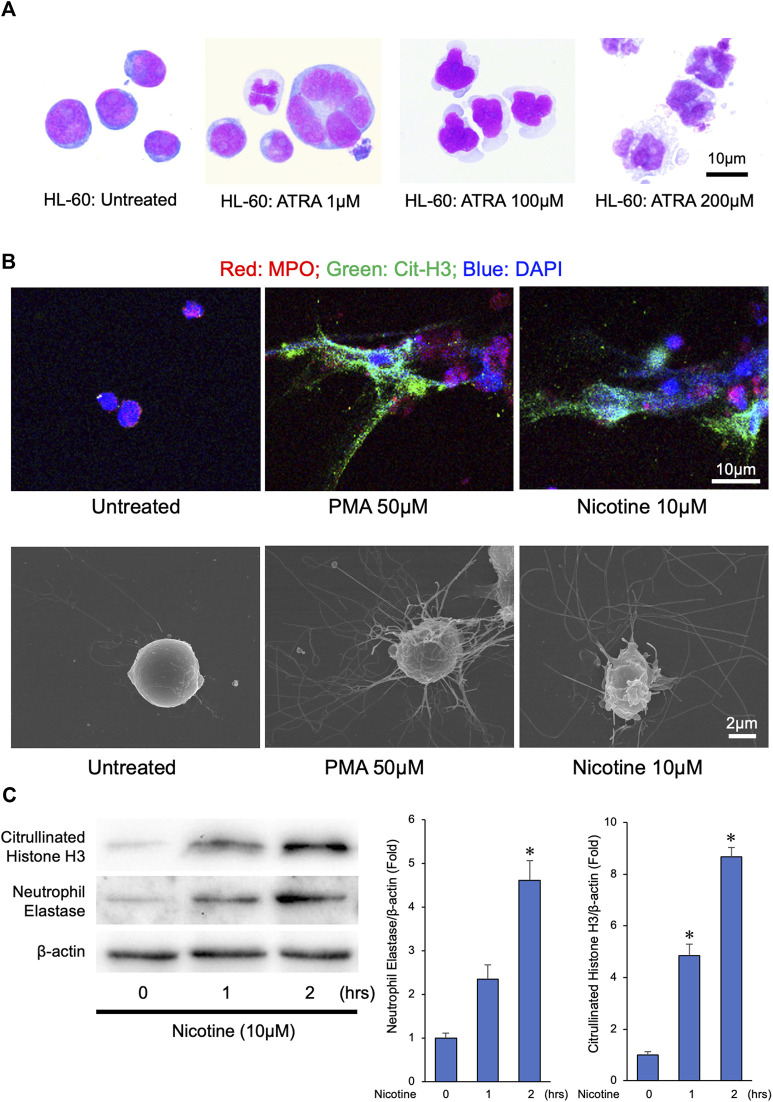
NETs were released from differentiated HL-60 in response to phorbol 12-myristate 13-acetate (PMA) or nicotine stimulation. **(A)** Human HL-60 cells were differentiated into neutrophil-like cells by treating HL-60 cells with ATRA (200 µM) for 72 h **(B)** NETs were induced by PMA (50 µM) or nicotine (10 µM) administration to differentiated HL-60 cells. Upper panels: Representative confocal immunofluorescence images of differentiated HL-60 cells with or without PMA or nicotine administration stained for myeloperoxidase (MPO, red), citrullinated histone 3 (Cit-H3, green), and DNA (DAPI, blue). Lower panels: Representative scanned electron microscopic images of the differentiated HL-60 cells with or without the PMA or nicotine administration. **(C)** Left panel: Representative immunoblot images of neutrophil elastase, citrullinated Histone H_3_, and β-actin. *Right panels*: Densitometric analysis of immunoblots. *p < 0.05 vs. 0-h, *n* = 3 in each group. All data are expressed as mean ± SEM.

Previous reports have shown that the nicotine induces NETs from an isolated human and mouse neutrophil leukocytes (Hosseinzadeh, Thompson, Segal and Urban, 2016; Lee et al., 2017). To establish an experimental system to obtain great amounts of NETs, we determined whether nicotine could induce NET formation from a differentiated HL-60 cells. To this end, we differentiated the human HL-60 cells into neutrophil-like cells by treating HL-60 cells with 200 µM of ATRA for 72 h (Figure 1A). Treatment with 50 µM of PMA, a well-known inducer of NETs, to neutrophil-like cells could effectively induce NETs (Figure 1B and Supplementary Figure S1). Similarly, treatment with 10 µM of nicotine to neutrophil-like cells resulted in the induction of NET formation (Figure 1B and Supplementary Figure S1). Consistently, immunoblot analyses revealed that the treatment with 10 µM of nicotine to neutrophil-like cells caused Histone H_3_ citrullination (Figure 1C). Furthermore, the abundance of NE, one of the major components of the NETs, was induced by the addition of nicotine (Figure 1C). These results suggest that the treatment with nicotine to neutrophil leukocytes promotes Histone H_3_ citrullination, thereby provoking NET formation, which results in the enhancement of NE release to the extracellular space.

### Neutrophil extracellular traps attenuate autophagosome formation and enhance inflammasome activity in macrophages

**FIGURE 2:**
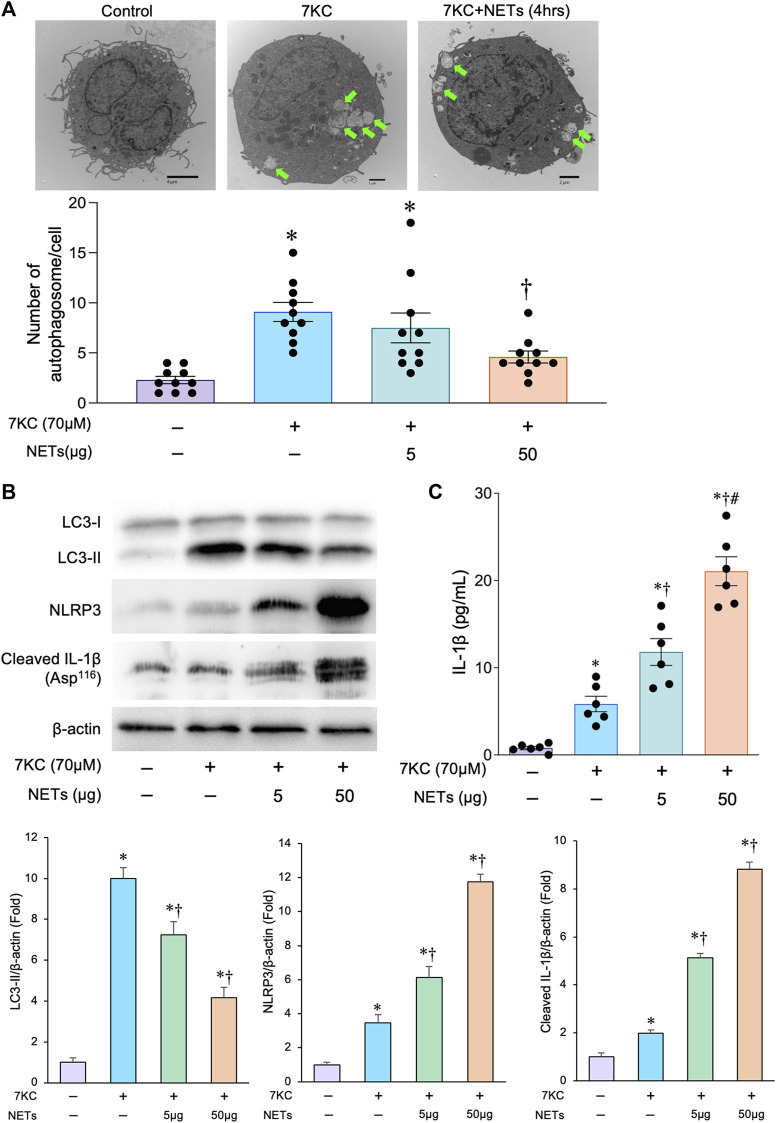
Effects of 7KC and NETs on macrophage autophagy *in-vitro*. **(A)** Upper panels: Representative transmission electron microscopic images of autophagosomes (arrows) in the macrophages differentiated from THP-1 cells with and without the administration of 7KC and or NETs. Lower panel: Quantitative analysis of the number of autophagosomes with and without the administration of 7KC and or NETs. *p < 0.05 vs. control group (Purple bar), ^†^ *p* < 0.05 vs. 7KC-treated group (Blue bar), *n* = 10 in each group. Data are expressed as dot plots and bars, with lines indicating the mean ± SEM. **(B)** *Upper panel*: Representative immunoblot images of LC3, NLRP3, cleaved IL-1β, and β-actin. Lower panels: Densitometric analysis of immunoblots. *p < 0.05 vs. control group (Purple bar), ^†^ *p* < 0.05 vs. 7KC-treated group (Blue bar), *n* = 3 in each group. All data are expressed as mean ± SEM. **(C)** Macrophages differentiated from the THP-1 cells with and without the administration of the 7KC and or NETs were analyzed for IL-1β secretion in the supernatants by the ELISA. *p < 0.05 vs. control group (Purple bar), ^†^ *p* < 0.05 vs. 7KC-treated group (Blue bar), ^#^ *p* < 0.05 vs. 7KC + NETs (5 μg)-treated group (Green bar), *n* = 6 in each group. Data are expressed as dot plots and bars, with lines indicating the mean ± SEM.

To analyze the effect of NETs on autophagosome formation in the macrophages, we conducted a couple of experiments in which the NETs released from neutrophil-like cells were added to the macrophages derived from human THP-1 cells. To mimic the role of macrophage autophagy in the pathological status of atherosclerosis, we used 7KC, an atherosclerotic lesioned oxysterol that promotes oxidative stress, inflammation, and autophagy, as an inducer of autophagosome formation (Liao et al., 2012; Ito et al., 2021). When macrophages were treated with a 7KC, the number of autophagosomes in the macrophages were increased, which was confirmed by a transmission electron microscopic examination (Figure 2A). Immunoblot analyses showed that the 7-KC treatment significantly increased the accumulation of LC3-II protein in the macrophages (Figure 2B), suggesting that the 7KC significantly promotes autophagosome formation in the macrophages. The addition of NETs significantly reduced the 7KC-induced autophagosome formation in a concentration-dependent manner as evidenced by the number of autophagosomes observed *via* transmission electron microscopy (Figure 2A). Immunoblot analyses consistently demonstrated that the treatment with a NETs significantly decreased the amount of LC3-II that was enhanced by a 7KC (Figure 2B). Treatment of macrophages with a 7-KC increased the expression of inflammasome components such as NLRP3 and cleaved IL-1β, and the addition of NETs further increased the amount of these inflammasome components in a concentration-dependent manner (Figures 2B,C). These results indicate that the NETs suppress autophagosome formation and enhance the inflammasome activity induced by a 7KC in the macrophages.

### Suppression of neutrophil extracellular traps formation resulted in the attenuation of atherosclerotic plaques in high-fat diet-fed *ApoE* ^*−/−*^mice

**FIGURE 3:**
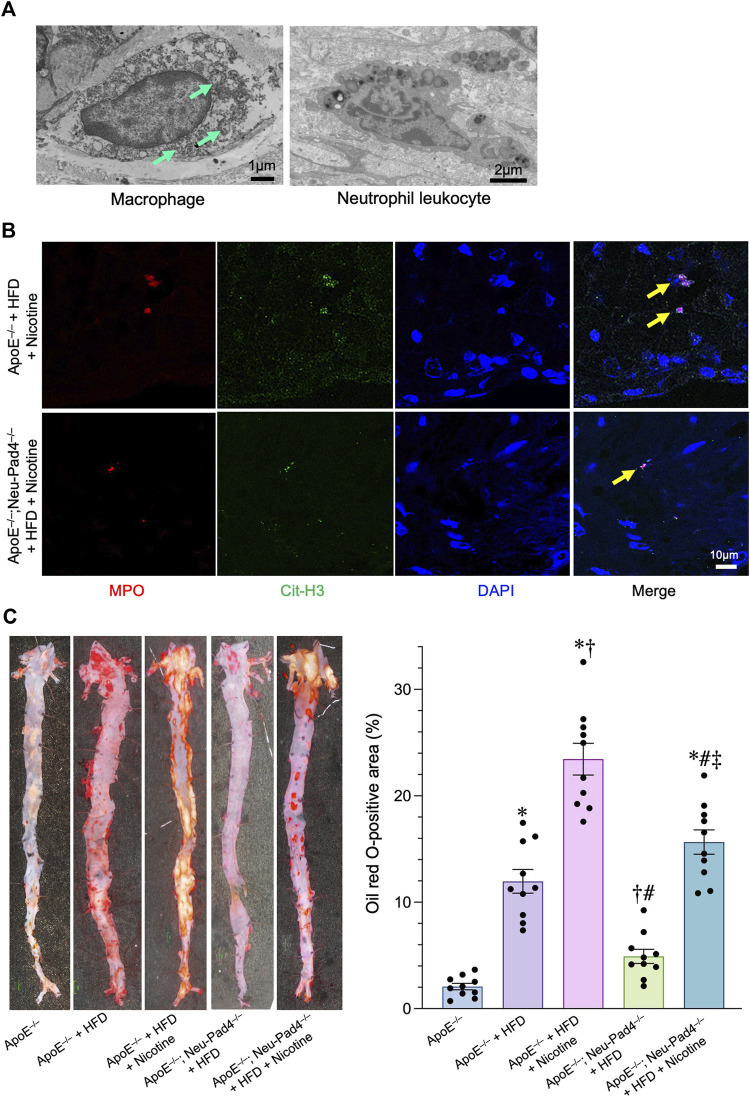
Role of NETs in the progression of atherosclerosis in high-fat diet (HFD)-fed *apolipoprotein E* knockout (*ApoE* ^*−/−*^) mice. **(A)** Upper left panel: A representative transmission electron microscopic image of autophagosomes (arrows) in the macrophage derived from the atheroma of HFD-fed *ApoE* ^*−/−*^mice. Upper right panel: A representative transmission electron microscopic image of the neutrophil leukocyte derived from the atheroma of HFD-fed *ApoE* ^*−/−*^mice. **(B)** Representative confocal immunofluorescence images of aortic sections from the indicated mice stained for myeloperoxidase (MPO, red), citrullinated histone 3 (Cit-H3, green), and DNA (DAPI, blue). **(C)** Right panel*:* representative images of the aorta *en face* derived from HFD-fed *ApoE* ^*−/−*^mice, *ApoE* ^*−/−*^; *Neu-Pad4* ^*−/−*^mice, or untreated *ApoE* ^*−/−*^mice, stained with Oil Red O. Right panel*:* quantification of aortic *en face* plaque area in HFD-fed *ApoE* ^*−/−*^mice, *ApoE* ^*−/−*^; *Neu-Pad4* ^*−/−*^mice, or untreated *ApoE* ^*−/−*^mice, as shown in the representative images. *p < 0.05 vs. Wild-type (Blue bar), ^†^ *p* < 0.05 vs. *ApoE* ^*−/−*^+ HFD (Purple bar), ^#^ *p* < 0.05 vs. *ApoE* ^*−/−*^+ HFD + Nicotine (Pink bar), ^‡^ *p* < 0.05 vs. *ApoE* ^*−/−*^ *; Neu-Pad4* ^*−/−*^+ HFD (Green bar), *n* = 10 in each group. Data are expressed as dot plots and bars, with lines indicating the mean ± SEM.

Histopathological analyses of atherosclerotic lesions of the HFD-fed *ApoE* ^*−/−*^mice revealed the localization of both the macrophages containing numerous autophagosomes and neutrophil leukocytes in the atherosclerotic plaques, suggesting that both cells would be contributing to the development of atherosclerotic lesions (Figure 3A and Supplementary Figure S2A). To investigate the effect of NETs on atherosclerosis *in vivo*, we generated neutrophil-specific *Pad4* knockout mice (*ApoE* ^*−/−*^ *; Neu-Pad4* ^*−/−*^) by crossing *Pad4* ^*fl/fl*^ mice with *Tg-Mrp8-Cre* mice and then crossing them with an *ApoE* ^*−/−*^mice. Both the *ApoE* ^*−/−*^ *; Neu-Pad4* ^*−/−*^mice and *ApoE* ^*−/−*^mice were fed with a HFD and given nicotine to induce NET formation *in vivo* (Supplementary Figure S2B). NETs could be detected in atherosclerotic lesions from HFD-fed *ApoE* ^*−/−*^mice and *ApoE* ^*−/−*^ *; Neu-Pad4* ^*−/−*^mice treated with nicotine (Figure 3B). Feeding of HFD increased the serum low-density lipoprotein cholesterol (LDL-C) level in *ApoE* ^*−/−*^mice (Table 1). On the contrary, neither neutrophil-specific *Pad4* ablation nor nicotine administration significantly changed the LDL-C level in the serum of experimental mice (Table 1). When nicotine was administered to the HFD-fed *ApoE* ^*−/−*^mice, the area of the atherosclerotic lesions was markedly enlarged (Wang et al., 2017). On the other hand, the area of the atherosclerosis was significantly smaller in *ApoE* ^*−/−*^ *; Neu-Pad4* ^*−/−*^mice fed with a HFD than in *ApoE* ^*−/−*^mice, regardless of whether the nicotine was administered or not (Figure 3C). These results indicate that the nicotine treatment increases the size of atherosclerotic lesions in a HFD-loaded *ApoE* ^*−/−*^mice, possibly through promoting the NETs formation.

**TABLE 1:** The serum lipid profile.

	Total cholesterol (mg/dl)	LDL-C (mg/dl)	Triglyceride (mg/dl)
ApoE^−/−^ (*n* = 10)	545.19 ± 40.80	309.34 ± 57.93	221.41 ± 48.66
ApoE^−/−^ + HFD (*n* = 10)	1064.12 ± 64.15*	1020.81 ± 89.09*	193.57 ± 28.31
ApoE^−/−^ + HFD + Nicotine (*n* = 10)	1207.80 ± 117.76*	1008.80 ± 113.24*	199.82 ± 26.95
ApoE^−/−^; Neu-Pad4^−/−^ + HFD (*n* = 10)	1142.86 ± 104.95*	971.31 ± 105.94*	216.84 ± 30.93
ApoE^−/−^; Neu-Pad4^−/−^ + HFD + Nicotine (*n* = 10)	1095.79 ± 99.54*	997.13 ± 102.44*	209.30 ± 47.23

*p < 0.05 vs. ApoE^−/−^.

LDL-C, low-density lipoprotein cholesterol.

### Neutrophil extracellular traps suppress Beclin-1-associated phosphatidylinositol 3-kinase activation by promoting Beclin-1 phosphorylation

**FIGURE 4:**
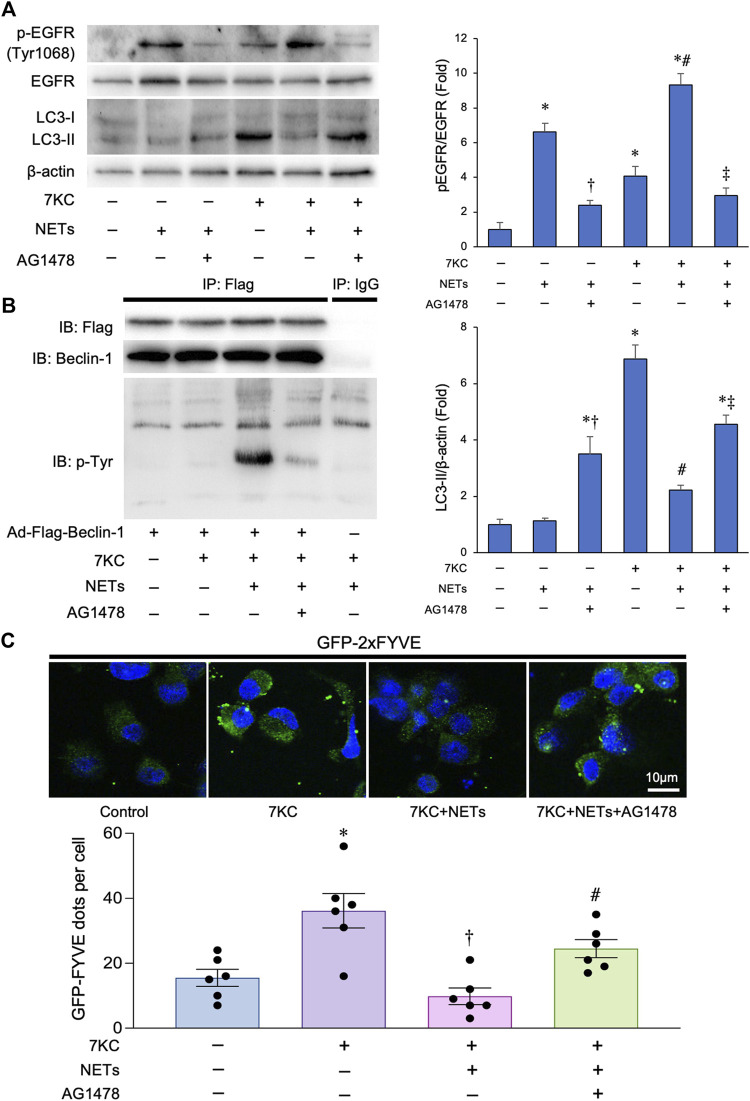
Effects of NETs for Beclin 1 phosphorylation and Beclin 1-associated PI3-K activation in macrophages. **(A)** Left panel: Representative immunoblot images of LC3, EGFR, phospho-EGFR (Tyr1068), and β-actin. Right panels: Densitometric analysis of immunoblots. *p < 0.05 vs. control group, ^†^ *p* < 0.05 vs. NET-treated group, ^#^ *p* < 0.05 vs. 7KC-treated group, ^‡^ *p* < 0.05 vs. 7KC + NET-treated group, *n* = 3 in each group. All data are expressed as mean ± SEM. **(B)** Macrophages derived from the THP-1 cells were treated with a 7KC after Ad-Flag-Beclin-1 transduction and then treated with or without the NETs or AG1478, a potent EGFR tyrosine kinase inhibitor. 48 h after the transduction, lysates were extracted for co-immunoprecipitation with phospho-Tyrosine-specific antibody or control IgG, followed by probing with Beclin-1 antibody. Representative images are shown. **(C)** Membrane-associated phosphoinositide 3-kinase (PI3-K) assay *in-situ*. Macrophages derived from the THP-1 cells were treated with a 7KC after Ad-GFP-2×FYVE transduction, and then treated with or without NETs or AG1478. *Upper:* Representative images of GFP-2×FYVE dots are shown. Lower*:* Quantitative analysis of the number of GFP-2×FYVE dots is shown. *p < 0.05 vs. control group (Blue bar), ^†^ *p* < 0.05 vs. 7KC-treated group (Purple bar), ^#^ *p* < 0.05 vs. 7KC + NETs-treated group (Pink bar), *n* = 6 in each group. Data are expressed as the dot plots and bars, with the lines indicating the mean ± SEM.

It has been shown that the NE, a major component of NETs, mediates ligand-dependent EGFR activation (DiCamillo et al., 2002; Kuwahara et al., 2006). To investigate the effect of NETs on EGFR of the macrophages, we added NETs to the cultured macrophages, and found that the addition of NETs promoted the phosphorylation of EGFR in the macrophages (Figure 4A). This reaction was significantly suppressed by the treatment with 10 μM of AG1478, a potent EGFR tyrosine kinase inhibitor. We next evaluated whether the NETs could phosphorylate Beclin-1 based on the previous finding that activated the EGFR phosphorylates tyrosine residues of Beclin-1, which decreases its function and suppresses the autophagy. To this end, we conducted immunoprecipitation assays using a macrophage-derived lysates overexpressing Flag-Beclin-1 with an anti-Flag tag-conjugated magnetic beads and immunoblotted with anti-tyrosine phosphorylation antibody and found that the tyrosine residues of Beclin-1 were markedly phosphorylated when the NETs were added. Co-administration of the AG1478, a potent tyrosine kinase inhibitor with NETs resulted in the attenuation of Beclin-1 phosphorylation (Figure 4B). To evaluate the effect of NETs on Beclin-1-dependent PI3 kinase activity, macrophages were transduced with the Ad-GFP-2×FYVE, a Phosphatidylinositol 3-phospate probe. Treatment with a 7KC significantly increased the number of GFP-2×FYVE dots in the macrophages. Conversely, co-treatment with a NETs significantly decreased the number of GFP-2×FYVE dots. On the other hand, the inhibition of EGFR activity with AG1478 administration significantly increased the number of the GFP-2×FYVE dots (Figure 4C). Altogether, these data suggests that the NETs inhibit the kinase activity of Beclin-1-dependent PI3 kinase activity possibly through phosphorylating the tyrosine residues of Beclin-1 in the macrophages.

### Neutrophil extracellular traps negatively regulate autophagosome–lysosome fusion through upregulating Rubicon expression

**FIGURE 5:**
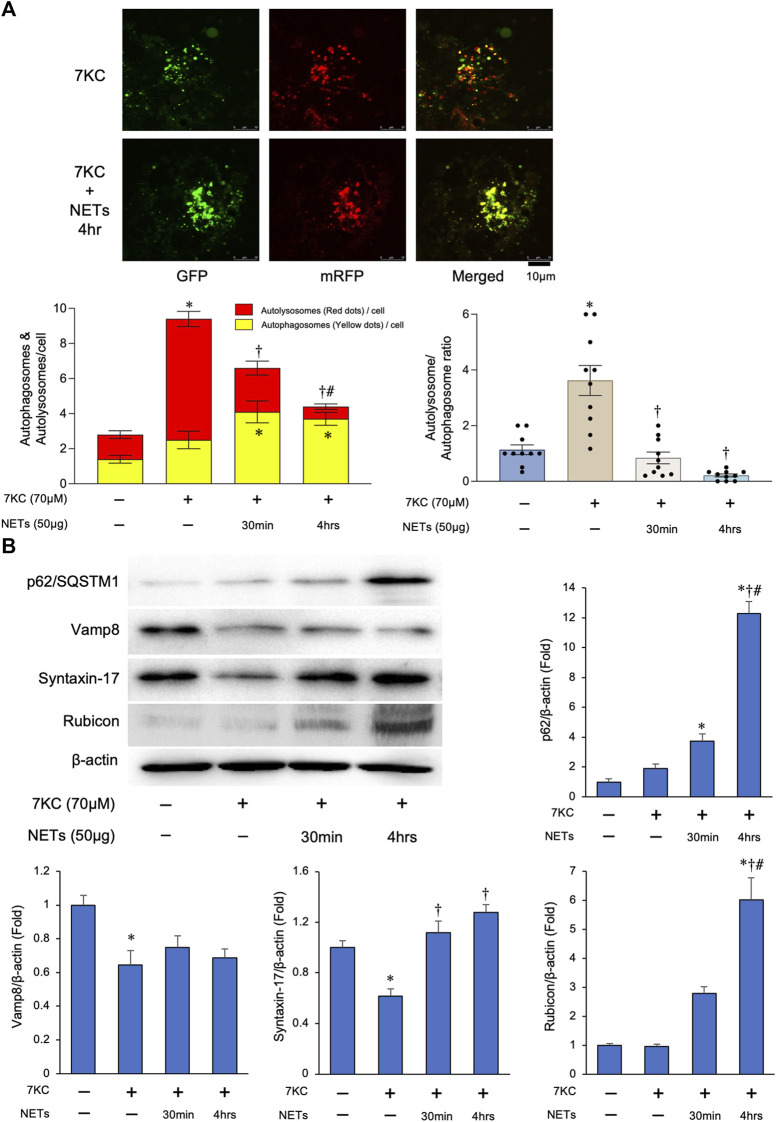
NETs negatively regulate autophagosome–lysosome fusion through upregulating Rubicon expression. **(A)** Autophagy flux assay. Macrophages derived from the THP-1 cells were treated with a 7KC after Ad-tf-LC3 transduction and then treated with or without the NETs. Upper panels*:* Representative images of the fluorescent LC3 puncta. Puncta with a yellow color in the merged images indicate the autophagosomes, and puncta with red color in the merged images indicate autolysosomes. Left lower panel: Quantitative analysis of the mean number of autophagosomes represented by the yellow puncta in the merged images and autolysosomes represented by the red puncta in the merged images per cell is shown. *p < 0.05 vs. control group, ^†^ *p* < 0.05 vs. 7KC-treated group, ^#^ *p* < 0.05 vs. 7KC + NETs (30 min)-treated group, *n* = 10 in each group. Data are expressed as the bars, with the lines indicating the mean ± SEM. Right lower panel*:* Quantitative analysis of the mean autophagosome/autolysosome ratio per cell is shown. *p < 0.05 vs. control group (Blue bar), ^†^ *p* < 0.05 vs. 7KC-treated group (Brown bar), *n* = 10 in each group. Data are expressed as dot plots and bars, with lines indicating the mean ± SEM. **(B)** Left upper panel: Representative immunoblot images of the p62/SQSTM1, Vamp8, Syntaxin-17, Rubicon, and β-actin. Right and Lower panels: Densitometric analysis of immunoblots. *p < 0.05 vs. control group, ^†^ *p* < 0.05 vs. 7KC-treated group, ^#^ *p* < 0.05 vs. NET (30 min)-treated group, *n* = 3 in each group. All data are expressed as mean ± SEM.

To investigate the effect of NETs on autophagosome and lysosome fusion reaction in the macrophages (i.e., autophagic flux), we conducted an autophagic flux assay using the macrophages transduced with Ad-tf-LC3. Merged images demonstrated that the increase of the red puncta by treating with a 7KC, was significantly greater than that in the yellow puncta in the macrophages as compared to the untreated one (Figure 5A). On the other hand, persistent treatment (4 h) of NETs with a 7KC significantly suppressed the number of red/yellow puncta ratio in the macrophages (Figure 5A). In addition, there was a significant accumulation of the p62/SQSTM1, a selective substrate for autophagy, by a persistent treatment of NETs with a 7KC in the macrophages (Figure 5B), suggesting that the NETs inhibit autophagic flux in the macrophages. To further investigate how to suppress the autophagic flux by a treatment with NETs in the macrophages, the expression levels of representative proteins involved in the autophagic flux were evaluated by immunoblotting. Among the SNARE proteins required for the fusion of autophagosomes and lysosomes, the level of Vamp8 was not changed by the addition of the NETs, while the level of Syntaxin-17 was slightly increased by a treatment with the NETs (Figure 5B). On the other hand, the expression of Rubicon, a protein that forms a complex with Beclin-1 for inhibiting the autophagosome/lysosome fusion, was increased markedly (Figure 5B).

Collectively, all these results indicate that the suppression of autophagic flux in the macrophages by a NETs is possibly mediated through the upregulation of a Rubicon by the treatment with NETs.

## Discussion

**FIGURE 6:**
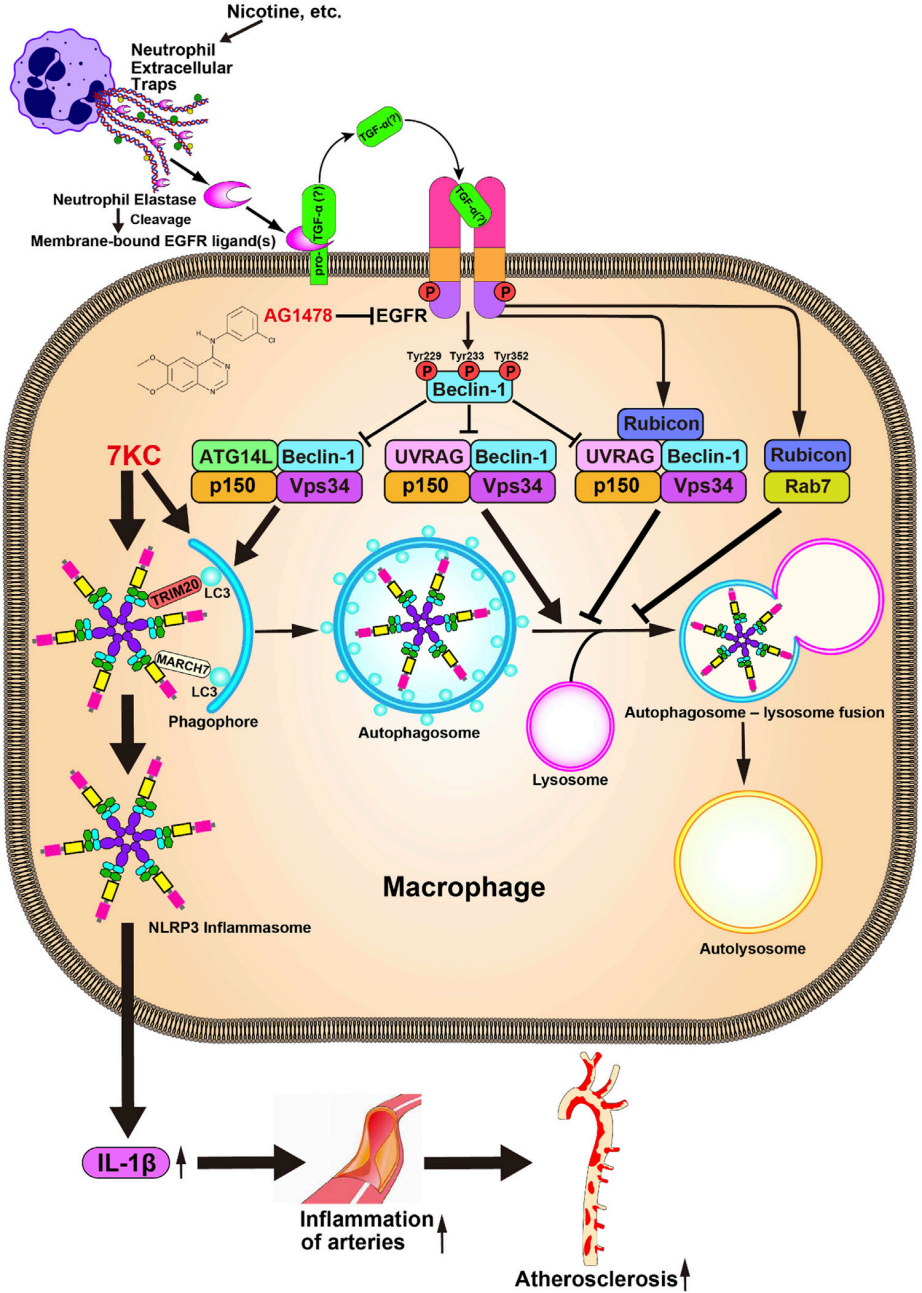
Proposed model for the deterioration of atherosclerosis by NETs. NETs induced by the various stimuli, including the nicotine, promote EGFR activity possibly through facilitating conversion of a membrane-bound EGFR ligand such as TGF-α, to its active soluble form by its proteolytic activity. Activated EGFR phosphorylates Beclin-1, thereby suppressing the Beclin-1-dependent PI3 kinase activity, which results in the suppression of an autophagy machinery. Attenuation of the autophagy activity causes enhancement of the inflammasome activity, which results in the aggravation of the atherosclerosis.

In the present study, we found that the NETs induced by nicotine, a common pro-atherosclerotic agent, negatively regulate autophagy in the macrophages, which causes an activation of the inflammasome activity. We also found that the NETs formation is critically associated with the atherogenesis in the HFD-loaded *ApoE* ^*−/−*^mice. Finally, we revealed that the suppression of autophagy by NETs is mediated through a tyrosine phosphorylation of Beclin-1, and increased expression of Rubicon possibly through an EGFR activation in the macrophages (Figure 6).

Although it has been shown that the NETs play an important role in promoting the atherosclerosis (Warnatsch et al., 2015), the cause of NETs induction other than the microbe infection has not been well investigated. In the present study, we found that the treatment with a nicotine to neutrophil leukocyte could induce NETs formation, which may support the epidemiological association of smoking habits with the development of atherosclerosis.

As mentioned above, we hypothesized that the NETs may worsen atherosclerosis by inhibiting autophagy machinery, thereby enhancing inflammasome activity in the macrophages. Here we show that the administration of NETs activates the EGFR, thereby promoting the phosphorylation of Beclin-1 in its tyrosine residues. Increasing lines of evidence suggest that the NE releases the soluble EGFR ligands, thereby activating the EGFR-mediated intracellular signaling (Jin et al., 2021). In cultured keratinocytes, the application of NE facilitates cell proliferation by promoting the conversion of a membrane-bound pro-TGF-α to its active soluble form by its proteolytic activity, thereby enhancing the EGFR activity (Meyer-Hoffert et al., 2004). Thus, it should be reasonable that the NETs promote EGFR phosphorylation. Indeed, we found that NET administration facilitates the phosphorylation of both EGFR and Beclin-1 at tyrosine residues, which is abolished by co-administration with EGFR tyrosine kinase inhibitor in the macrophages. Concurrently, we found that the level of Rubicon was markedly increased in the macrophages by treatment with NETs. However, its detailed mechanism remains unknown. Li et al. (2021) recently reported an intriguing finding that the EGFR suppresses autophagy by upregulating the Rubicon in podocytes (Li et al., 2021). Based on these findings, NET-derived NE would promote EGFR activation, thereby facilitating both Beclin-1 phosphorylation and Rubicon expression, which in turn inhibit autophagy machinery in the macrophages. Thus, in the present study, we could add a novel finding regarding the role of EGFR as an autophagy modulator, which contributes to the progression of atherosclerosis.

One of the critical tasks of autophagy is removing dangerous objects in the cells such as damaged mitochondria and excessively activated inflammasome, which plays an important role in the progression of the atherosclerosis by enhancing a sterile inflammation triggered by the damage-associated molecular patterns (Liao et al., 2012; Razani et al., 2012; Ito et al., 2021). Specifically, the inflammasomes are degraded by a selective type of autophagy, termed as “precision autophagy,” by recognizing with the specialized receptors such as TRIM20 and MARCH7 (Kimura et al., 2015; Yan et al., 2015). Indeed, we recently revealed that the NLRP3-inflammasome is decomposed by a TRIM20-mediated precision autophagy in the 7KC-treated macrophages (Ito et al., 2021). In this study, we found that inhibition of autophagy by NETs contributes to the maintenance of the inflammasome activity, suggesting that the NETs may have a crucial role in inhibiting the precision autophagy.

Our experiments have several potential limitations. Our study demonstrated that the area of atherosclerotic plaque significantly decreased in a nicotine-treated HFD-fed *ApoE* ^*−/−*^ *; Neu-Pad4* ^*−/−*^mice compared to those in HFD-fed *ApoE* ^*−/−*^mice. This result indicates that the nicotine-mediated atherogenesis is partially mediated through the NETs formation. However, there should be other mediators that promote NET formation, which results in atherosclerosis. To verify the role of NETs in animal models, proteinase 3-deficient (*PR3* ^*−/−*^) and/or *NE* ^*−/−*^mice would be crossed with *ApoE* ^*−/−*^mice because deleting these enzymes might more effectively abrogate NET formation (Warnatsch et al., 2015). In addition, to validate whether NET-mediated suppression of macrophage autophagy plays an important role for developing atherosclerosis *in vivo*, loss-of-function animal models such as macrophage-specific Atg5 knockout mice should be crossed with *ApoE* ^*−/−*^ *; Neu-Pad4* ^*−/−*^mice. Similarly, cell experiments should be conducted using primary macrophages derived from macrophage-specific Atg5-knockout mice. Furthermore, as 7KC promotes not only autophagy but also oxidative stress and apoptosis, a line of experiments using pure pro-autophagic inducers should be conducted to evaluate the effect of NETs on macrophage autophagy in the presence of pathological conditions other than atherosclerosis. It is also insufficient to detect Beclin-1 phosphorylation by blotting with total phospho-tyrosine antibody. Ideally, the effect of NETs on Beclin-1 should be examined by blotting with a specific antibody that can detect Beclin-1 phosphorylation in Tyr229, Tyr233, and Tyr352 (Wei et al., 2013). From this perspective, further studies are needed in the future to increase the reliability of the current findings.

In conclusion, our current study revealed that the NET-mediated EGFR–Beclin-1 signaling in the macrophages promotes atherogenesis by accelerating the inflammasome activities, which is mediated through suppressing the autophagy. This suggests that the NETs-mediated EGFR–Beclin-1 signaling could be a novel candidate for developing the anti-atherosclerotic agent.

## Data Availability

The raw data supporting the conclusions of this article will be made available by the authors, without undue reservation.
