# Supplementary material for: Neutrophil extracellular traps-mediated Beclin-1 suppression aggravates atherosclerosis by inhibiting macrophage autophagy
Source: Front Cell Dev Biol. 2022 Jul 18;10:876147. doi: 10.3389/fcell.2022.876147 (PMC9340257; doi:10.3389/fcell.2022.876147)
Supplement: Supplementary file 1 [file Presentation1.PDF]

Supplementary Figure 1

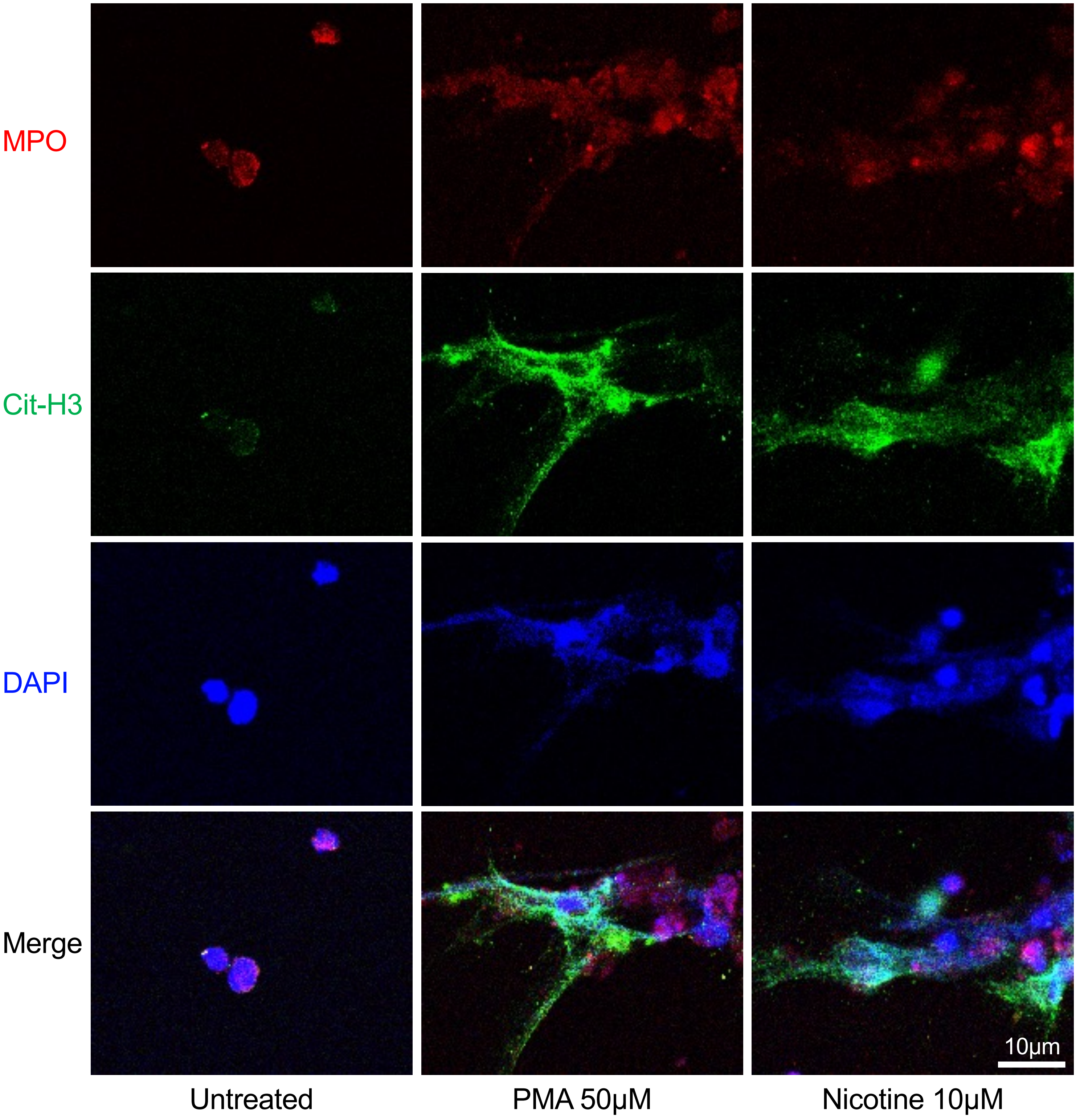

Representative confocal immunofluorescence images of differentiated HL-60 cells with or without the PMA or nicotine administration stained for myeloperoxidase (MPO, red), citrullinated histone 3 (Cit-H3, green), and DNA (DAPI, blue). Merged images are shown in Figure 1B.

Supplementary Figure 2

A

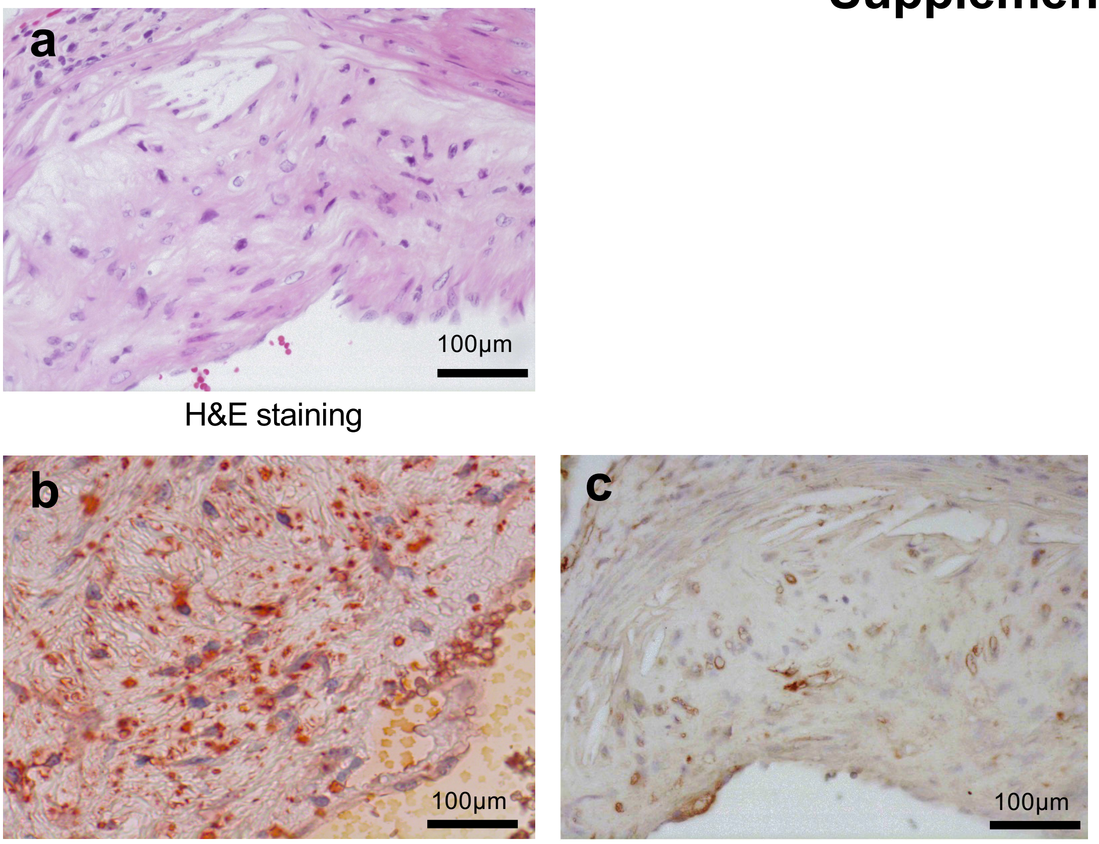

B

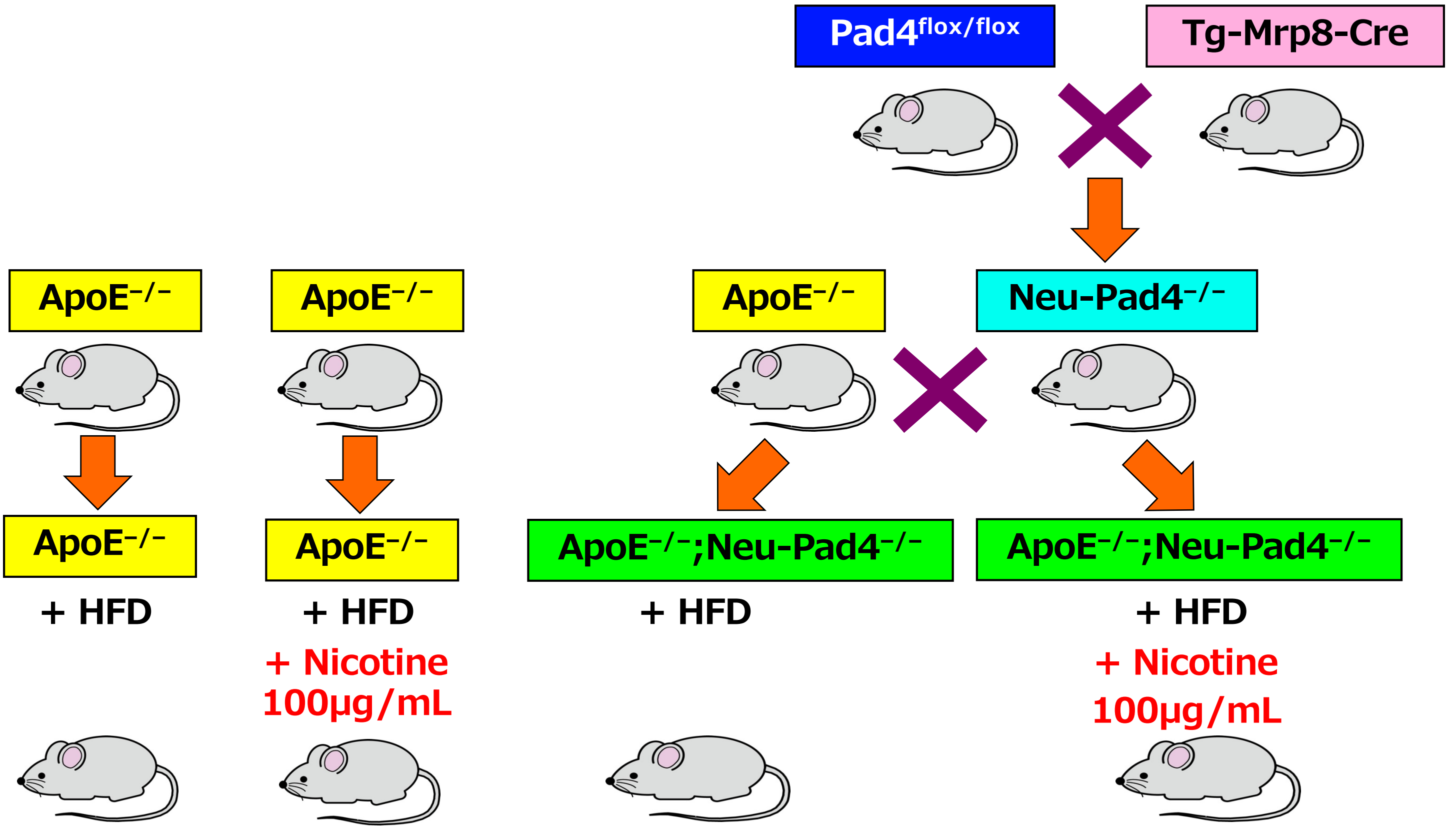

A. A representative image of H&E staining (a), anti-CD11b (a marker of macrophages) immunostaining (b), and anti-MPO (a marker of neutrophil leukocytes) immunostaining (c) in atheroma derived from HFD-fed *ApoE*$^{-/-}$ mice. **B.** Scheme of the experimental design for evaluating the effect of NETs on atherosclerosis. *ApoE*$^{-/-}$ mice, *ApoE*$^{-/-}$*; Neu-Pad4*$^{-/-}$ mice, or wild-type mice were fed a HFD for 20 weeks with or without treatment with a nicotine (a NETs inducer).
